# Retraction: Designing of an mRNA vaccine against high-risk human papillomavirus targeting the E6 and E7 oncoproteins exploiting immunoinformatics and dynamic simulation

**DOI:** 10.1371/journal.pone.0323771

**Published:** 2025-04-30

**Authors:** The PLOS One Editors

The *PLOS One* Editors retract this article [1] due to concerns about compromised peer review that call into question the validity of the reported results. We regret that the issues were not identified prior to the article’s publication.

All authors did not agree with the retraction.
